# Mississippi Valley Dental Society

**Published:** 1879-05

**Authors:** 


### Proceedings of Societies.

### MISSISSIPPI VALLEY DENTAL SOCIETY.

*Discussions at the Thirty-Fifth Annual Meeting.*

*Continued from page 176.*

### AFTERNOON SESSION.

The society met at half past two, p. m., and was called to order by the president, Dr. Hunter.

The subject under discussion at the hour of adjournment, "Chemical processes that occur in the mouth," was passed.

On motion of Dr. J. Taft the speakers were limited to fifteen minutes for an opening address, and five minutes for each subsequent speech.

The second subject on the programme, "The effects of systemic diseases upon the dental organism," was then taken up for discussion.

The members manifesting the usual reluctance to open the subject, Dr. J. Taft spoke as follows: I was a little at a loss to know just what was meant by this subject, but I presume it is the effect of general disease upon the teeth, and possibly the adjacent parts. Systemic diseases vary very much in character. Some strike at the muscular system, others at the nervous, others at the vascular or circulatory system, others at the nutritive. Those affections that effect the nutritive functions more especially impair the entire organization, and the teeth will suffer correspondingly with other parts of the body. If, as we heard this morning, they are not endowed with vitality, then this proposition is not true; but that they are supplied with the nutritive function and nerve instrumentality, is just about as plain to me as that the rays of the sun are shining here upon the floor, and I don't know how any man who has given attention to the teeth for any considerable time, can arrive at any other conclusion. During the time of development it is much more true that they will fail to be properly built up if there is defective nutrition, and are by that means impaired forever. Any deterioration, caused by failure of nutrition, any breach made at that time, will involve the whole, and is never wholly repaired although there may be some improvement. The life force is sometimes so deteriorated and impaired that the pulps simply die without any indications of decay whatever. Nervous affections are often found centering themselves in the teeth. Other nerves are better shielded, especially from thermal changes, than those of the teeth, these being quite impressible, as we all experience, when we take very hot or cold substances in the mouth, and bring them in contact with the teeth. The teeth make a louder outcry from thermal changes than any other part. You may put your hand in a very cold fluid and no pain is experienced, but apply the same to the living teeth and there is pain.

I apprehend we can include in the discussion of this subject the dental periosteum; and that is subject to systemic diseases in a great variety of ways. Anything that will produce an abnormal condition of the periosteum anywhere is liable to produce it in the periosteum of the teeth, and any injury to the periosteum anywhere will be markedly felt upon the periosteum of the teeth. Cutaneous diseases effect the teeth at any time, but more certainly and effectually during the time of development, than at any other time. Measles, small-pox, scarlatina, occurring at the time of the development of the teeth sometimes affect them in a most marked manner, and perhaps, upon the whole, more injury is done to the teeth by systemic disease of this kind, at this time of life than by all others combined.

Dr. Rawls stated that he introduced the subject more for the purpose of getting an expression of opinions as to Rigg's disease so-called than for anything else.

Dr. Meredith stated that this was one of the subjects left over from last year's meeting. According to the statement of the subject we simply look to the tooth itself, and see what effect is produced by certain diseases and there we stop. As we look at the teeth, the most glaring effect is, perhaps, that of atrophy, and I think it would not be wandering far from the subject to speak of the different kinds of dental atrophy and the manner of filling teeth. Another effect is where the whole structure of the tooth itself is frail, and seems unable to resist the action of the fluids of the mouth.

Dr. Smith gave an instance of an aggravated case of Rigg's disease, in the dental infirmary, where the condition of the patient was that of ænemia; and yet the parts were being restored again.

Dr. Rawls—I think these cases must be simply of local lesion, influenced. more or less, by the condition of the general system. If the patient is ænemic we may have a very rapid deterioration. The deposition of matter of any character, either bony or soft tissue, will depend, of course, altogether upon the amount of vitality in the system to build it up. I remember the question of the reproduction of the gum and alveolar process, that came up at the Indiana State Dental Association, at Fort Wayne, and Dr. Taft and Dr. Atkinson, of New York, were very enthusiastic upon the reproduction of the tissues around the teeth. Dr. Atkinson stated his process, giving reasons for thinking the tissues would be reproduced, by stating that a pocket was formed between the gums and the tooth for the deposition of embryonic material. But we may have all these conditions and protection be wanting. We can't have this embryonic tissue remain there unless the parts are protected from the action of anything that would interrupt its normal growth. And each tissue must produce its like, No tissue will develop another and dissimilar tissue. Periosteum must be developed of itself, and the other parts of themselves. If this tissue is perfect then there are little loops of blood vessels thrown out into this embryonic tissue, and unless that is thoroughly protected not only the embryonic tissue goes, but the little blood vessels go too, but if we can thoroughly protect the parts and then work upon the system in such manner as to give it all the vitality possible, and then surround the parts with such local conditions as will favor reproduction, we may have it, not only of one tissue, but of all. Medication in such cases is entirely out of the question, except to stimulate the parts to a reaction, not only locally but generally. A great many people have a habit of using very strong remedies in such cases, and in doing so destroy tissues as rapidly as they are built up.

Dr. Smith—Is the origin systemic or local?

Dr. Rawls—So far as you report your case I can't believe that it is systemic.

Dr. Smith--What becomes of the tissue? Is it absorbed or dissolved?

Dr. Rawls—So far as I understand the case I think it simply a disintegrating process brought about by the contact of foreign material and irritating substances that intercepts the circulation of the parts. (Dr. Rawls here explained his views by means of diagrams on the board).

Dr. Smith agreed with Dr. Rawls as to the overtreatment of cases. The great difficulty is in taking care of the new growth. He used a small portion of sponge saturated with glycerine and a very small amount of carbolic acid to prevent putrefaction in the sponge.

Dr. Taft—Did you give any systemic treatment?

Dr. Smith—I did not, because the patient designs going away very soon. I should have given her some preparation of quinine and iron, if she were going to remain, in order to build up the red corpuscles of the blood, which seem almost entirely wanting.

Dr. Taft thought the case mentioned by Dr. Smith, almost entirely due to local irritation, which was evidenced by reparation of the parts, and advised careful local treatment in preference to sending the patient to a physician, who in nine cases out of ten would do him no good, because the difficulty was local.

Dr. Osmond—I have found that persons afflicted with that disease are in the habit of discarding vegetables almost entirely, and living on beefsteak and bread. I think it entirely useless to use local treatment in such a case. The only remedy is to use a vegetable food and drink freely of milk. Medical treatment will always fail in these cases.

Dr. Meredith—I would like to hear a discussion of the subject of recession of the gums. That is the method of losing the teeth in after life, and it is becoming very prevalent in young persons, of late years. I think it one of the diseases of hyper-nutrition. A case was sent to me, from the East, not long ago, in which it appeared that even the root was covered with enamel. Another case is that of wearing away the tips of the teeth, the incisors and cuspids especially, so that the dentine is exposed, and this is found as early as the age of twenty-five and thirty, so that the teeth require tipping and filling, or copper-toeing as it is sometimes called. I think there must be some change of late years in the treatment of teeth which causes this, and if there is any method of treatment which will retard it, I think it a good time to speak of it.

Dr. Taylor—I think probably we all agree that through systemic influences the dental organs are very much injured or benefitted during the formative process. If the mother is in bad health of course the teeth of the off-spring will be poor. You can't make a good organ out of poor material. If the mother is in an emaciated condition, with the red corpuscles of the blood almost exhausted, where do you get the lime necessary for the formation of dental or even osseous structure. I suppose you have all noticed that if you put a good and a bad tooth in the same acid, the poor one will be much more rapidly dissolved than the good one.

Dr. Taylor proceeded to cite some remarkable cases of recession of the gums which had come under his treatment, which he explained by means of illustrations on the blackboard. In one case the gum had enlarged very much, and resisted all his treatment until he procured a needle and pricked the gum around the teeth, perhaps in a dozen places. The effect was very fine and at the time of reporting, he had every indication that the case would soon be off his hands, but he did not think they would ever grow back as far around the neck of the tooth as originally.

Dr. Morgan thought Dr. Taylor's case simply a local affection, or else it presented a remarkable instance of systemic disease cured by local treatment. He said in cases where there are violent fever attacks, the teeth will be affected. There will be imperfect dentition a portion of the lime salts washing out very soon.

Especially will those persons who are treated actively with mercury, labor under this disease. All the skin diseases may be included as causes. Typhoid fever is the worst disease, however. I don't know whether I understood Dr. Taft rightly or not (I was mighty sleepy during a part of the time, I confess) but I understood him to say that if the nervous tissues were diseased it did not necessarily influence the teeth. Now typhoid fever is preeminently of that nature, and yet you have a condition of the fluids of the mouth which actually tends to dissolve the enamel, and where a patient has been confined with typhoid fever for three or four weeks, you will find the teeth darkened and generally softened; and you will find too in the course of a year or two that it will go on and a large number of approximal fillings will have to be made.

Dr. Templeton, of Pittsburgh, cited the case of a little girl about fifteen years old who was brought to his office and who had several teeth operated upon. She came back in about six months and he found that several of the fillings he had previously inserted were loose and there was decay around them. The girl had lost about one-half her hair, and upon inquiry he learned that she had had scarlet fever.

Dr. Rawls—I don't believe with Dr. Taft that nineteen-twentieths of all such cases can be cured by local treatment properly applied; at least so far as my locality is concerned. In the swampy districts of the country we have cases much more frequently than in the city, and a case has never been permanently cured by local treatment. I defy any man to produce a case so cured. It is a condition bordering on the death of the tooth.

Dr. Taft said the great difficulty was over medication. It was true, that connected with syphilitic conditions those cases are very hard to manage, but he had seen them brought back to health and the teeth made firm and solid, not one case in a hundred was effected to the extent mentioned by Dr. Rawls.

Dr. Templeton said he had practiced for eight years in a district as malarious as possible and had seen the gums loose from the teeth all round, and he had arrested the process, restored them to health and made them as sound as a baby's gums.

Dr. Rawls—From local treatment?

Dr. Templeton—Yes sir; although I would have used systemic treatment sometimes, but you know the people don't like to take systemic treatment from a dentist. Subject passed.

The third subject "Influences upon Second Dentition by Derangement of the First" was then discussed as follows:

Dr. Taft opened the discussion by enumerating the diseases of first dentition and giving some of their results.

Dr. Morgan said that after the temporary teeth are all developed a diseased condition might be set up in the molars that would produce the same symptoms as when the temporary teeth are erupting, even to producing convulsions.

Dr. Keely—The temporary teeth have their mission to perform as well as the permanent set; and one great source of derangement is the loss of the temporary teeth before the time for the permanent teeth to appear. I have heard gentlemen say they could destroy the pulps of the temporary teeth and fill them just as they would the permanent teeth, but I

have never been fortunate enough to do that. I think the temporary molars more important teeth to preserve in order to insure a good permanent set. I always preserve them if possible, and if I can't do that I destroy the pulps and preserve the roots.

Dr. Taft desired to enter a caution against the destruction of the pulps even of temporary teeth.

Dr. Smith thought there was a limit to the saving of the first teeth. He thought the alarming injury to the second teeth by the premature extraction of the first, largely imaginary. He had seen children who had had all their first teeth removed, with very perfect second teeth.

Dr. Berry thought it best to preserve the temporary teeth.

Dr. Rawls said that the development of the jaw is undoubtedly retarded to some extent by the extraction of the deciduous teeth. The permanent teeth would not be apt to come in regularly.

Dr. Osmond—Whenever you have a diseased temporary tooth which you have to remove it is always necessary in order to prevent irregularity in the permanent teeth, to remove the sound one next to it. I believe, however, in keeping the temporary teeth just as long as possible.

Dr. Clayton said it had been his observation that more irregularity is caused by leaving the roots of temporary teeth in the mouth than by removing them, but admitted it was desirable to save the live temporary teeth.

Dr. Morgan believed that the temporary teeth might be taken out without injury.

Dr. Clayton cited the case of a little child, who had always been very healthy but latterly had shown signs of convulsions; he wanted to know if that was caused by a retarded tooth.

Dr. Taft said it possibly was and recommended incision of the gums. Subject passed.

On motion the meeting adjourned until nine a. m., Thursday morning.

SECOND DAY—MORNING SESSION.

Thursday morning, March 6th, 1879, the society met pursuant to adjournment.

The third subject, which was under consideration at the hour of adjournment the previous evening, was farther discussed by Drs. Mollyneaux, Taft, Dennis, Keely and Rosenthal, and was then passed.

The fourth subject "The New Dental Departure," was then taken up for discussion.

Dr. J. Taft opened the discussion. He said he desired to draw out some expressions of opinion from those present. He did not attribute as much importance to the subject as a good many do; he thought it was making a very large claim on a very small basis. It is said that gold is not the best thing to save teeth. In one sense that is true; in another it is as false as it can be. Tin is sometimes better than gold.

The new departure says, "in proportion as the teeth need to be saved, gold is the worst material used." What is meant by the phrase, "as teeth *need* saving?" I presume it means in proportion as teeth are difficult to save, gold is the worst article. I think the better common sense of the profession would hardly indorse that statement.

The second article says, "some have faith in contours; others have faith in separation." Now these are placed in antithesis, but there is no propriety in doing so. The model for every case in filling is as far as possible to restore the original contour of the tooth, and I don't know anybody that don't have faith in separation.

They say also that "failure in operation is mainly due to defective manipulation." Now we all know that failure is often due to defective structure in the teeth, and from various other causes. Very poor fillings often arrest decay, whereas the best of fillings and the best of manipulations fail sometimes because of other unfavorable conditions.

Time was here called on the doctor.

Dr. Smith then read a paper on this subject.

Dr. Taft thought the electric current had no more to do directly with decomposition of dentine than has the heat evolved, but said it must not be left out of view that both heat and the electric current did promote chemical change in the substance round about them, but so far as the direct influence was concerned neither had anything to do with the decomposition of the teeth.

Dr. Leslie—Although there has been a good deal said to-day, no one comes forward with a demonstration that proves anything. Eminent men differ essentially in their conclusions on this subject. Bridgeman was the first experimenter in England, and Chase is the most distinguished experimenter in this country. It is the accepted theory of all electricians now, that the current of electricity is the result of chemical action. Now Bridgemen discovered that both gold and dentine are positive; that is that when they are brought together there is no action. Tin, however is negative and therefore an unfit material for filling. Now I have failed to find that gold is incompactible with tooth bone, and I don't think it makes any difference as to leakage if the electrical current exists. The fluid on the surface is sufficient; and these men admit that there may be dissolution at this point Dr. Chase says that when you fill a tooth with gold, you have got a battery.

Dr. Smith read some selections from Prof. Chase's writings and commented upon them.

Dr. Dravens—Dr. Palmer does not agree altogether with Drs. Flagg and Chase. He believes that if a man could fill a tooth with gold well, there is no justification in his taking amalgam instead if the patient is willing and able to pay for it. I don't think tin and gold combined work well, but I have used tin alone, with good results, and amalgam also. But tin used in combination with gold will become black and deteriorated in substance like a slag. Gutta percha filling will not leak, if not put in too hot, or worked down with an instrument that is too hot.

If the advocates of the new departure were honest in their belief, I could respect them, but Dr. Flagg, at least, I have good reason to believe, is actuated by mercenary motives. I met S. S. White not long ago, and asked him what Dr. Flagg was making out of his new departure—referring to his progress—and White said "money." Now I think that the profession have advertised the manufactures of these individuals long enough, and for my part I do not intend to lend my share in advertising Dr. Flagg's productions any more. Applause!

Dr. Sage thought that filling failed at the very point of the servical margin where the instrument fractures the edge. He had found that decay was not so likely to occur in those teeth which are easily accessible, so that the gold can be perfectly impacted. He therefore thought that the inaccessibility of the cavities had much to do with the failure of operations.

Dr. Leslie said there was nothing new in the new departure. Fillings have been used for over forty years.

On motion the society adjourned until nine a. m. Friday morning.

THIRD DAY—MORNING SESSION.

The society met pursuant to adjournment. The minutes of the previous meeting were read and approved.

The subject under discussion at the hour of adjournment—"The new Departure" was further discussed as follows:

Dr. Cassidy—We know now nearly as much of matter and force as I fear we will ever know. We will know a great deal more of the effects which may be produced by force and matter, but I am afraid we will never know the ultimate nature of either. We know something about heat and electricity; but yesterday we were told that electricity is a force of nature, distinct and independent in itself. We have also heard that heat and light are each distinctive from the other. And to-day we are told that there is but one force. We think we know a great deal about matter, but along comes a man, eminent in the scientific world, and makes us smile by declaring that all tangible things are composed of one element. Another man says it is hydrogen and declares that on the authority of experiment. Experiment has proven that force is manifested by motion of any kind. The electric current is not developed in solids without antecedent motion of some kind, mechanical, vital or chemical. If a current of electricity be passed through a solid, that solid is not effected in any way, physically or chemically. But if an electric current passes through a liquid capable of conducting it, that liquid is decomposed; if not capable of conducting it, it is not decomposed. Chemical solution of a solid must precede galvanic action. Electricity never causes solution of a solid except by fusion. The electric cnrrent is established when any solid undergoes solution. But is solution or the solvent action increased by the presence of the electric current. It may be in some cases, but I don't think it is in the action of a solvent upon tooth bone by the mere presence of gold, or any other material.

Dr. Taft—You mean, directly?

Dr. Cassidy—Yes sir, directly. I don't think the presence of gold will increase the activity of the molecular motion of any solvent, acting upon tooth bone. There is the electric current established, but that is simply the effect of the previous solution of the solid, and that electric current does not establish a galvanic battery; it ceases the moment the solvent is exhausted. That is according to well known experiment. But I confess that I think the alchemists, when they sought for the Alcahest or spirit of matter, knew as much about it as we do, or as I fear we will ever know.

Dr. Howe—The prominent point connected with the new departure to me has not been the theoretical presentation of the case, but the fact that the plastic material is the fittest material to be employed provided it has the element of durability. The most notable thing in regard to the osteo-plastic material is that it absolutely fits the cavity. I am for the survival of the fittest—that which fits the cavity. That is what gives promise of the best results.

Dr. Smith—Dr. Thompson, of Kansas, says that an essential quality for a desirable filling is adhesion to the tooth. Oxy-chloride of zinc adheres to the wall of a cavity where it is perfectly clean; and I think the new filling must possess that quality of adhesion. The difficulty is that we are all talking about perfect fillings while the new departure men are all the time talking about leaky fillings. It is not fair to say that these men claim that there is *always* chemical action set up between gold and dentine, and Dr. Chase says that one filling is just as good as another so long as there is no chemical action or dissolution.

Dr. Jay—Then if that be true, gold is just as good a material as any other.

Dr. Hunter—I understand him to say so.

Dr. Jay—Then why does Dr. Flagg say it is the worst filling?

Dr. Hunter—I don't suppose Dr. Chase should be held responsible for Dr. Flagg's statements.

Dr. Jay—If electrical action is produced by dissolution of particles of matter; and if there is nothing to induce that dissolution it amounts to nothing.

Dr. J. Taft—Who are the men in the profession that give countenance to this new departure? Is it such men as Crydon, Palmer, Dwinelle, Watt, Atkinson, McKellops, C. R. Butler, Morgan, Field, Hunt, Jennings, etc.?

On the other hand, does not the entire quack element of the profession support and practice the new departure, and do they not in justification of their mischievous practice point to Drs. Flagg, Chase & Co? Is there no significance in this?

Is it to be supposed for one moment that such men as those first named, have never given any thought or study to the distinctive points of the alleged new practice? Is it not well known that many of them have gone vastly farther into all these subjects than any or all of the originators of the new departure.

If the thought, study and discussion, which have been given to this subject, had been as industriously employed in some other direction, far more good would have resulted to the profession and to the public--for instance, about the structure and function of parts and organs, upon the abnormal condition to which they are subject, and the best methods of treating these conditions, and the best agents to be used in such treatment. how much does the average dentist know? Would it not be better to devote more time to these, and such things that lay at the foundation, rather than to devote so much upon theories that have at best but a shadowy support by experiment or demonstration? Is the time most profitably spent in such discussion?

The sixth subject, "Novel Dental Operation," was next taken up, and the time for its discussion limited to thirty minutes.

Dr. Osmond asked the experience of the profession in regard to the new practice introduced by Dr. Richmond, not long ago, of putting a gold cap on roots of teeth.

Dr. French said he had a limited experience. Dr. Richmond gave a clinical operation in his office, and while he took rather kindly to the operation, he thought Richmond was one of the most bungling operators he had ever seen.

Dr. Hunter—Was he a red-haired or a dark-haired man?

Dr. French—Red.

Dr. Osborne had doubts as to whether Richmond was the inventor or not. He had on two occasions given him the dates of his patent, which he was utterly unable to find.

Dr. Howe—I have a tooth filled by Dr. Richmond. Drs. Hunter, Smith and Read were present, and saw the operation performed. We each paid him ten dollars for instruction. The tooth has done very well. It is firm, but it annoys me very much, because it was not finished well. He filled off the wrong cusp.

Dr. Hunter was then called upon to explain the proceess under discussion. He responded in an interesting manner, but not sufficiently in detail to be of value to the profession.

Dr. Templeton gave his experience with Dr. Richmond, which was by no means flattering to the last named gentleman.

Dr. Will. Taft—There is one point in this thing that has not been mentioned, and that is the recession of the gums. Dr. Richmond had three of these operations in his own mouth, and about every one of the teeth the gums had resceded. About the central incisor the gums were absorbed very much, and the tooth was so loose that you could have removed it with the fingers.

Dr. Sage—I have noticed that same peculiarity about the teeth in Dr. Richmond's mouth. It looked to me as though there was either necrosis of the edge of the alveolar process, or a deposit of dark salivary calculus.

At this point the discussion was interrupted, and a committee was appointed, consisting of Drs. Morgan, J. Taft, Cassidy and Smith, to draft resolutions on the death of Prof. McQuillan, of Philadelphia.

The discussion was resumed. Dr. Cravens said that periostitis had given him most serious trouble the past winter, and he desired information from others on the subject.

Dr. Jay believed that the increased prevalence and virulence of malarial affections has had much to do with the remarkable increase in periostitis. In these cases he did not believe that topical remedies amounted to anything.

Dr. J. Taft said that much good was often effected by depletion in any of the ordinary ways.

Dr. Morgan—It is easy to tell the channels through which remedies perform their proper office; but how do they perform it?

Not a single word in explanation of the *modus operandi* can be given. That is one of the things. We don't understand and perhaps never are to understand. All we know is, that their administration brings about certain results under certain conditions. When there is irritation the red corpuscles being larger than the white began to crowd in and hang on to each other and the walls of the capillary vessels and by-and-by they actually dam them up like drift in a stream, and we have status of the blood and inflammation as a result. Now any procedure that will enable a free flow in these vessels will relieve. Sometimes it can be done by contracting the vessels and they can be actually *squez* out. Sometimes it can be done by letting the blood out. Sometimes it can be done by depletion, sometimes by an astringent, sometimes by a stimulant.

Dr. Rawls—Dr. Morgan makes a mistake when he says that the red blood corpuscles attach themselves to the walls of the blood vessels. It is the white. I deny that we don't understand any thing about the action of these remedies. We know the physical characteristics of the changes that take place after the application of these remedies. For instance, an astringent causes soft tissue to contract. How it does that we don't exactly know. There has been a very great want of understanding in relation to the modes of these simple topical remedies for inflammation. We see it every day almost in persons who have suffered at the hands of dentists, who do not know how these applications operate and at what time to apply them. Dentists are concerned more perhaps with inflammation than with any other disease of the body. In fact we can't have a disease of any kind without inflammatory action. And dentists are concerned more with topical applications than with systemic remedies. Iodine acts not only as an astringent, but as a stimulant.

Dr. Morgan said he thought that under the microscope the red corpuscles would be found to adhere to the walls of the vessels.

Dr. Osmond said that unless systemic remedies were used in connection with topical remedies in cases of inflammation, the latter would be utterly useless. The association at this point was closed.

